# Relationship between time-varying status of reflux esophagitis and *Helicobacter pylori* and progression to long-segment Barrett’s esophagus: time-dependent Cox proportional-hazards analysis

**DOI:** 10.1186/s12876-020-01418-5

**Published:** 2020-08-15

**Authors:** Genki Usui, Tomohiro Shinozaki, Toyohisa Jinno, Kazutoshi Fujibayashi, Teppei Morikawa, Toshiaki Gunji, Nobuyuki Matsuhashi

**Affiliations:** 1 Department of Diagnostic Pathology, NTT Medical Center Tokyo, 5-9-22 Higashi-gotanda, Shinagawa-ku, Tokyo, 141-8625 Japan; 2 Department of Information and Computer Technology, Faculty of Engineering, Tokyo University of Science, Tokyo, Japan; 3 Center for Preventive Medicine, NTT Medical Center, Tokyo, Tokyo Japan; 4 Department of General Medicine, Juntendo University Hospital, Tokyo, Japan; 5 Department of Gastroenterology, NTT Medical Center, Tokyo, Tokyo Japan

**Keywords:** *Helicobacter pylori*, Eradication, Reflux esophagitis, Barrett’s esophagus, SSBE, LSBE, Endoscopy, Elongation, Time-dependent, Time-varying

## Abstract

**Background:**

Reflux esophagitis (RE) and absence of *Helicobacter pylori* (non-*H. pylori*) are considered to be associated with the progression to long-segment Barrett’s esophagus (LSBE). However, it is difficult to assess this association because RE and *H. pylori* status can change during follow-up. Additionally, the association between *H. pylori* eradication and LSBE remains unclear.

**Methods:**

A total of 11,493 asymptomatic Japanese subjects who underwent medical check-ups and were endoscopically diagnosed with short-segment Barrett’s esophagus (SSBE) between May 2006 and December 2015 were enrolled. The hazards of progression to LSBE were compared between time-varying RE and *H. pylori* infection/eradication by time-dependent multivariable Cox proportional hazards models.

**Results:**

A total of 7637 subjects who underwent additional medical check-ups after being diagnosed with endoscopic SSBE were analyzed. Subjects with RE and without current/past *H. pylori* infection were strongly associated with a higher rate of progression to LSBE (adjusted hazard ratio [HR]: 7.17, 95% confidence interval [CI]: 2.48–20.73, *p* < 0.001 for RE and non-*H. pylori* vs. non-RE and *H. pylori* groups). Subjects with *H. pylori* had a lower rate of progression to LSBE (adjusted HR: 0.48, 95% CI: 0.22–1.07, *p* = 0.07 for *H. pylori* vs. non-*H. pylori*). Hazards of progression to LSBE were still lower in the *H. pylori* eradication group than that of the non-*H. pylori* group (adjusted HR: 0.51, 95% CI: 0.18–1.46, *p* = 0.21).

**Conclusions:**

RE and non-*H. pylori* were associated with the progression to LSBE, considering the changes in exposures. *H. pylori* infection was associated with the prevention of the development of LSBE irrespective of RE. The environment preventive of the development of LSBE persists for at least a few years after *H. pylori* eradication.

## Background

Barrett’s esophagus (BE) is known as a premalignant lesion of esophageal adenocarcinoma (EAC) [1–3]. BE is classified into short-segment BE (< 3 cm) (SSBE) and long-segment BE (≧ 3 cm) (LSBE) based on the segment lengths of esophageal mucosa with columnar metaplasia [4]. LSBE, a higher-risk condition for the development of EAC than SSBE, is common in Western countries [5]. On the other hand, the lengths of most BEs in Asia are less than 3 cm [6, 7]. In Japan, more patients are endoscopically diagnosed with SSBE, some of which are known to have different risk factors than those with LSBE [8]. It is clinically essential to investigate the frequency and clinical characteristics of endoscopic SSBE in developing into LSBE or EAC. However, the natural history of endoscopic SSBE elongation remains unknown.

Reflux esophagitis (RE) and absence of *Helicobacter pylori* (non-*H. pylori*) are considered to be associated with SSBE elongation to LSBE [9]. *H. pylori* infection causes gastric atrophy and decreases gastric acid secretion, leading to a decreased prevalence of RE and BE [10, 11]. Past studies showed that *H. pylori* infection suppressed the elongation of SSBE (odds ratio [OR]: 0.71, 95% confidence interval [CI]: 0.44–1.15) [9]. However, it is difficult to accurately assess these associations because some RE subjects start or stop taking antacids, and some *H. pylori* infections are eradicated during follow-up. There have been no studies that investigate the associations referring to changes in RE and *H. pylori* status.

The association between *H. pylori* eradication and progression to LSBE remains unclear. *H. pylori* eradication is a useful treatment for reducing the risk of developing gastric cancer [12, 13]. However, some studies reported that *H. pylori* eradication increases gastric acid secretion, leading to an increased prevalence of RE [14]. Therefore, *H. pylori* eradication may also promote BE [15], which has not yet been confirmed.

There are two severe challenges in accurately estimating the association between RE and *H. pylori* status and progression to LSBE. First, it is challenging to collect clinical data several times over time. In particular, the assessment of RE and *H. pylori* status requires endoscopy and *H. pylori* tests, such as the serum anti-*H. pylori* IgG antibody test, which are not routinely conducted. The second is the analysis of time-varying exposures. In Cox proportional hazards regression models, exposures are usually fixed at baseline. However, especially in observational studies with long follow-up periods, exposures can often change during follow-up. Cox models with covariates fixed at baseline may mislead the true association by misclassifying exposures during the follow-up period.

Therefore, we conducted a retrospective cohort study aimed at investigating the association between RE and *H. pylori* status and progression to LSBE using medical check-up data collected over time. We used time-dependent covariates in the Cox models, which may estimate the accurate impact of RE or *H. pylori* infection and eradication on the progression to LSBE.

## Methods

### Study population and design

A total of 11,493 asymptomatic Japanese subjects who underwent the Early Disease Detection and Prevention program at NTT Medical Center Tokyo in Japan and who were endoscopically diagnosed with SSBE from May 2006 to December 2015 were enrolled. In this hospital, the Early Disease Detection and Prevention program is conducted with asymptotic subjects [8, 16, 17]. This program includes medical interviews about alcohol consumption, smoking habits, medical and treatment histories, physical and physiological examinations, blood tests including serum anti-*H. pylori* IgG antibody test, and esophagogastroduodenoscopy screening. Subjects with a history of esophagectomy or gastrectomy were excluded from the analysis. Subjects who did not undergo an additional program after being diagnosed with SSBE were also excluded. Our study protocol was approved by the institutional ethics committee.

### Data collection

Endoscopy specialists who did not know the subjects’ information performed esophagogastroduodenoscopy. The endoscopic diagnosis of RE and BE was conducted according to the Los Angeles classification system [18, 19], and Prague C & M Criteria [20]. In accordance with a previous multicenter prospective cohort study in Japan, the site of the esophagogastric junction was defined in our study as the proximal end of the mucosal folds continuous from the stomach or distal ends of the palisade vessels [21]. Endoscopically diagnosed BE is called endoscopic BE, columnar-lined esophagus or endoscopically suspected esophageal metaplasia [22–25]. When the distance between the circumferential extension of the gastric mucosal fold and the proximal edge was 1–3 cm or exceeded 3 cm, it was defined as an endoscopic SSBE or LSBE, respectively. After endoscopists conducted the endoscopic examinations, the most experienced endoscopist (N.M.) reviewed key images of all examinations. He conducted weekly endoscopic image reviews (3–12 key images per subject) throughout the study period to confirm the accurate diagnosis.

*H. pylori* infection was defined as *H. pylori* seropositivity and the presence of endoscopic atrophic gastritis (AG). Serological *H. pylori* status was assessed by using an enzyme-linked immunosorbent assay (Eiken Chemical, Tokyo, Japan). The seropositive antibody titer threshold for *H. pylori* infection was set at 10 U/ml. Its sensitivity and specificity were over 85% [26, 27]. *H. pylori* seronegative and endoscopically AG negative subjects were categorized as non-*H. pylori* infection, and the others were categorized as *H. pylori* infection. Subjects with *H. pylori* infection were classified into current *H. pylori* infection and *H. pylori* eradication groups. These categories were updated at each subject’s visit through the follow-up period.

Smoking habits were assessed using pack-years (packs of cigarettes per day multiplied by smoking years). Subjects were categorized into four groups according to the amount of alcohol consumption per week: nondrinker (< 40 g/week), light drinker (40–140 g/week), moderate drinker (140–280 g/week), and heavy drinker (> 280 g/week) [8, 16].

### Statistical analysis

The subjects were categorized into four groups according to the combinations of RE and *H. pylori* status (non-*H. pylori* or *H. pylori* infection). The probability of progressing to LSBE according to RE and *H. pylori* status at baseline was estimated using the Kaplan–Meier method. This analysis did not take into account both the change in RE and *H. pylori* status. In subsequent analyses, the following information was updated in a time-dependent manner: age, smoking, RE, medication (proton pump inhibitor (PPI) or histamine H2-receptor antagonist (H2RA)), and *H. pylori* status. The rate of progression to LSBE for each time-varying RE and *H. pylori* status was calculated using the person-years method and compared between the RE and *H. pylori* statuses through hazard ratios (HRs) estimated by multivariable Cox models with time-dependent covariates. In these models, the covariates, including RE and *H. pylori* status, were updated at a medical check-up. If a subject progressed to LSBE after a status change, this information contributes to event counts in the new status, while information about not progressing to LSBE by the progression time contributes to both the previous and the new statuses depending on her/his observed time. The association between *H. pylori* status (current infection or eradication) and progression to LSBE was also assessed by time-dependent Cox models. In model 1, subjects were categorized into two groups: non-*H. pylori* infection and *H. pylori* infection groups. In model 2, subjects were categorized into three groups: non-*H. pylori* infection, current *H. pylori* infection, and *H. pylori* eradication groups. Because the convergence of parameter estimates was not achieved in the initial Cox model that fully adjusted for possible risk factors, we carefully checked the convergence and estimates for every combination of covariates adjusted. Accordingly, we selected sex, age, smoking, hiatal hernia, RE, and medication (PPI or H2RA) to adjust for, as we found alcohol consumption did not predict the outcome in either univariable or multivariable models.

We conducted the statistical analysis by using SAS 9.4 (SAS Institute, Cary, NC) and EZR (Saitama Medical Center, Jichi Medical University, Saitama, Japan), which is a graphic user interface for R (The R Foundation for statistical computing, Vienna, Austria). EZR is an enhanced version of the R commander with a statistical function [28].

## Results

Among the 11,493 subjects who were diagnosed with endoscopic SSBE between May 2006 and December 2015, 192 subjects with a history of esophagectomy or gastrectomy were excluded. Among the remaining 11,301 subjects, 7637 subjects who underwent additional health checks by November 2018 were included in the analysis.

The baseline characteristics of the subjects with endoscopic SSBE are presented in Supplemental Table 1. At baseline, a total of 4067 subjects (53.3%) were negative for *H. pylori* infection, 2732 subjects (35.8%) were currently positive for *H. pylori* infection, and 819 subjects (10.7%) had a history of successful *H. pylori* eradication. A total of 3176 subjects (41.6%) were negative for RE and positive for *H. pylori* infection, 3020 subjects (39.5%) were negative for RE and *H. pylori* infection, 375 subjects (4.9%) were positive for RE and *H. pylori* infection, and 1047 subjects (13.7%) were positive for RE and negative for *H. pylori* infection. The characteristics of the subjects categorized according to baseline RE and *H. pylori* infection status are presented in Table 1.

**Table 1:** Differences in various parameters by RE and *H. pylori* status pattern at baseline

	RE(−)/*H. pylori*(+) (*n* = 3176)	RE(−)/*H. pylori*(−) (*n* = 3020)	RE(+)/*H. pylori*(+) (*n* = 375)	RE(+)/*H. pylori*(−) (*n* = 1047)
Age (years) (mean (SD))	54.5 (9.6)	47.3 (10.0)	52.9 (8.7)	47.7 (9.4)
Sex, men (%)	2618 (82.4)	2351 (77.8)	353 (94.1)	936 (89.4)
Smoking (pack-years) (mean ± SD)	14.8 (17.3)	10.3 (14.9)	20.0 (18.9)	12.4 (15.0)
Alcohol consumption				
Nondrinker (< 40 g/week) (%)	1185 (37.4)	1210 (40.1)	110 (29.3)	391 (37.3)
Light-drinker (40–140 g/week) (%)	848 (26.7)	846 (28.0)	90 (24.0)	266 (25.4)
Moderate-drinker (140–280 g/week) (%)	615 (19.4)	558 (18.5)	83 (22.1)	217 (20.7)
Heavy-drinker (≥280 g/week) (%)	524 (16.5)	406 (13.4)	92 (24.5)	173 (16.5)
Hiatal hernia (% positive)	642 (20.2)	939 (31.1)	138 (36.8)	425 (40.6)
PPI or H2RA (% positive)	77 (2.4)	76 (2.5)	8 (2.1)	15 (1.4)

*RE* Reflux esophagitis; *H. pylori Helicobacter pylori*; *SD* Standard deviation; *PPI* Proton pump inhibitor; *H2RA* Histamine H2-receptor antagonist

**Fig. 1:**
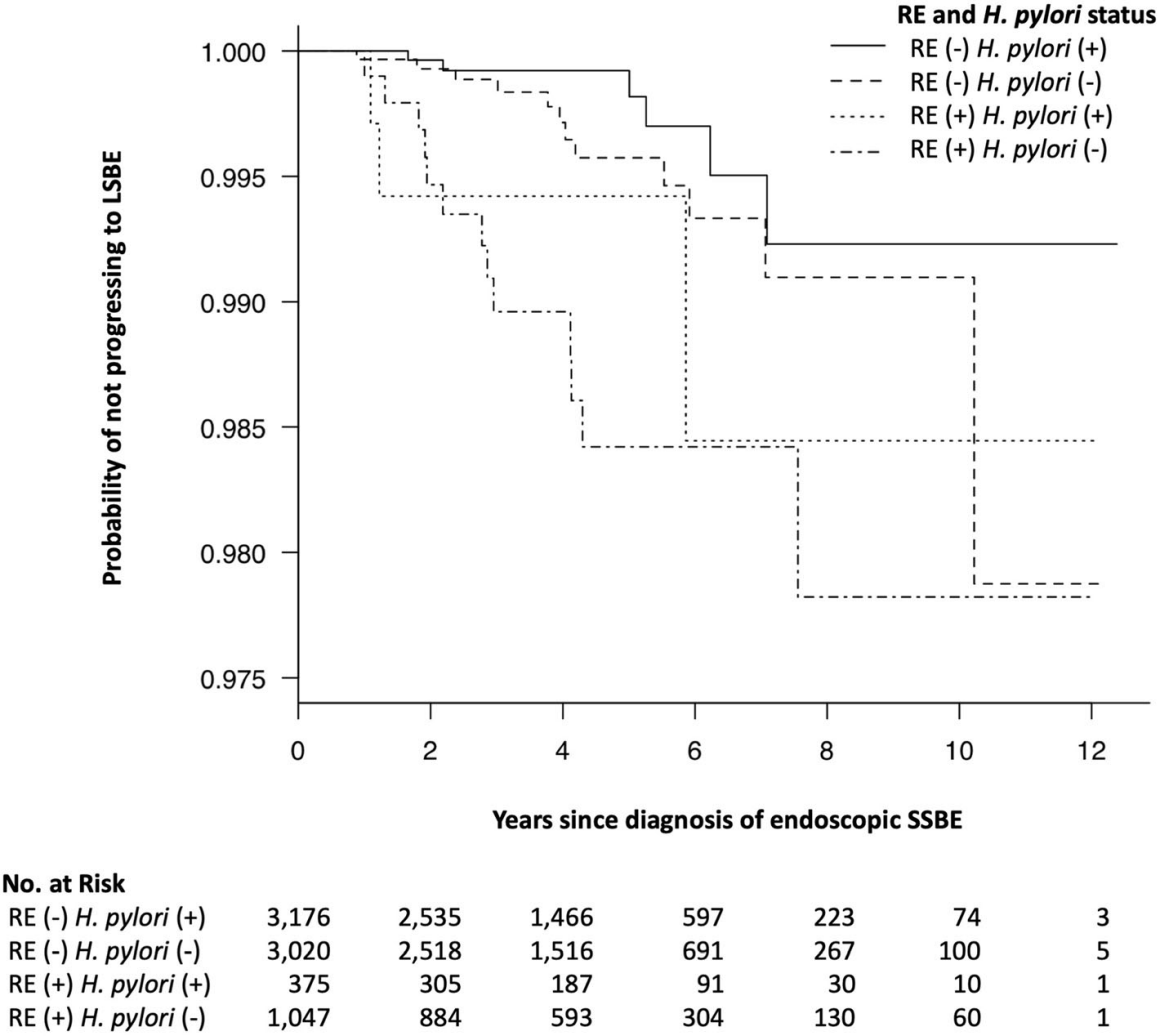
Kaplan–Meier estimates of the probability of not progressing to LSBE, according to RE and *H. pylori* status. Curves are shown for the probability of not progressing to LSBE according to RE and *H. pylori* status

The median follow-up period was 4.0 years. During the follow-up period, 34 subjects showed progression to LSBE. The Kaplan–Meier curves show the probability of not progressing to LSBE, according to RE and *H. pylori* status at baseline (Fig. 1). These curves suggested that the probability of progression to LSBE was lower in subjects without RE and with *H. pylori* infection than in the other groups. However, this analysis did not consider changes in RE or *H. pylori* status. In subsequent analyses, the covariates were time-dependently updated. Of the 7637 subjects with endoscopic SSBE, none had low- or high-grade dysplasia during the study.

During the follow-up period, the rate of progression to LSBE was 1.0 per 1000 person-years. The progression rate for each time-varying RE and *H. pylori* status was as follows: 0.4 (non-RE and *H. pylori* infection), 0.9 (non-RE and non-*H. pylori* infection), 2.2 (RE and *H. pylori* infection), and 2.8 per 1000 person-years (RE and non-*H. pylori* infection) (Table 2).

**Table 2:** The rate of progression to LSBE for each time-varying RE and *H. pylori* status

Time-varying RE and *H. pylor*i status	Person-years	Elongation of SSBE to LSBE	Rate (per 1000 person-years)
RE(−) /*H. pylori* (+)	12,889	5	0.4
RE(−) /*H. pylori* (−)	13,571	12	0.9
RE(+) /*H. pylori* (+)	1829	4	2.2
RE(+) /*H. pylori* (−)	4710	13	2.8

*SSBE* Short-segment Barrett’s esophagus; *LSBE* Long-segment Barrett’s esophagus; *RE* Reflux esophagitis; *H. pylori* Helicobacter pylori

Table 3 shows HRs of time-varying RE and *H. pylori* status for the progression to LSBE in the multivariable time-dependent Cox models. RE and non-*H. pylori* group and RE and *H. pylori* group were strongly associated with a higher rate of progression to LSBE (adjusted HR: 7.17, 95% CI: 2.48–20.73, *p* < 0.001 for RE and non-*H. pylori* vs. non-RE and *H. pylori* group; adjusted HR: 4.57, 95% CI: 1.26–16.63, *p* = 0.02 for RE and *H. pylori* vs. non-RE and *H. pylori* group). Non-RE and non-*H. pylori* infection groups were also associated with a higher rate of progression to LSBE but were imprecisely estimated due to limited event numbers (adjusted HR: 2.45, 95% CI: 0.86–7.00, *p* = 0.09 for non-RE and non-*H. pylori* vs. non-RE and *H. pylori* groups). In the multivariable Cox models with fixed covariates, the HR of RE and non-*H. pylori* for the progression to LSBE was attenuated (adjusted HR: 5.59, 95% CI: 2.04–15.36, *p* < 0.001 for RE and non-*H. pylori* vs. non-RE and *H. pylori* groups) (Supplemental Table 2). In addition, the HR of smoking for the progression to LSBE was lower in Cox models by baseline time-fixed covariate (adjusted HR (per 100 pack-years): 1.64, 95% CI: 0.29–9.11, *p* = 0.57) than that in the time-dependent Cox model (adjusted HR (per 100 pack-years): 3.66, 95% CI: 1.09–12.29, *p* = 0.04).

**Table 3:** Association between RE and *H. pylori* status pattern and the progression to LSBE adjusted by time-dependent covariate

	HR*	95% CI		*P*-value
RE and *H. pylori* status pattern				
RE(−) /*H. pylori* (+)	1.00	Reference		
RE(−) /*H. pylori* (−)	2.45	0.86	7.00	0.09
RE(+) /*H. pylori* (+)	4.57	1.26	16.63	0.02
RE(+) /*H. pylori* (−)	7.17	2.48	20.73	< 0.001
Male	1.18	0.41	3.45	0.76
Age (year)	1.05	1.01	1.09	0.01
Smoking (100 pack-year)	3.66	1.09	12.29	0.04
Hiatal hernia	3.13	1.49	6.58	0.003
PPI or H2RA	3.99	1.54	10.33	0.004

*Adjusted by time-dependent covariate (RE and *H. pylori* status pattern, age, smoking, and PPI or H2RA) and baseline time-fixed covariate (male and hiatal hernia)

*RE* Reflux esophagitis; *H. pylori Helicobacter pylori*; *LSBE* Long-segment Barrett’s esophagus; *HR* Hazard ratio; *CI* Confidence interval; *PPI* Proton pump inhibitor; *H2RA* Histamine H2-receptor antagonist

Table 4 shows HRs of time-varying *H. pylori* status for the progression to LSBE in the multivariable time-dependent Cox models. Model 1 showed an adjusted HR of 0.48 (95% CI: 0.22–1.07, *p* = 0.07 for *H. pylori* vs. non-*H. pylori*), which suggests a decrease in the rate of progression to LSBE by *H. pylori* infection. In model 2, the hazards of progression to LSBE were still lower than those of non-*H. pylori* group in the current *H. pylori* infection group (adjusted HR: 0.50, 95% CI: 0.19–1.34, *p* = 0.17) and *H. pylori* eradication group (adjusted HR: 0.51, 95% CI: 0.18–1.46, *p* = 0.21). In the multivariable Cox models with fixed covariates, HRs and their 95% CIs of *H. pylori* eradication for the progression to LSBE remained similar (Supplemental Table 3).

**Table 4:** Association between *H. pylori* status and the progression to LSBE adjusted by time-dependent covariate

	Multivariable model 1				Multivariable model 2			
	HR*	95% CI		*P*-value	HR*	95% CI		*P*-value
*H. pylori* status								
Absence of *H. pylori*	1.00	Reference			1.00	Reference		
*H. pylori* infection (Model 1)	0.48	0.22	1.07	0.07				
Current *H. pylori* infection (Model 2)					0.50	0.19	1.34	0.17
*H. pylori* eradication (Model 2)					0.51	0.18	1.46	0.21
Male	1.19	0.41	3.47	0.75	1.19	0.41	3.47	0.75
Age (year)	1.05	1.01	1.09	0.01	1.05	1.01	1.09	0.01
Reflux esophagitis	3.27	1.63	6.56	< 0.001	3.27	1.63	6.55	< 0.001
Smoking (100 pack-year)	3.67	1.10	12.26	0.03	3.67	1.10	12.29	0.03
Hiatal hernia	3.16	1.50	6.66	0.003	3.16	1.50	6.66	0.003
PPI or H2RA	4.00	1.54	10.36	0.004	4.00	1.54	10.35	0.004

*Adjusted by time-dependent covariate (*H. pylori* status, age, reflux esophagitis, smoking, and PPI or H2RA) and baseline time-fixed covariate (male and hiatal hernia).

*H. pylori Helicobacter pylori*; *LSBE* Long-segment Barrett’s esophagus; *HR* Hazard ratio; *CI* Confidence interval; *PPI* Proton pump inhibitor; *H2RA* Histamine H2-receptor antagonist

## Discussion

This is the first retrospective cohort study to investigate the association of RE and *H. pylori* with the progression from endoscopic SSBE to LSBE referring to changes in RE and *H. pylori* status. Progression to LSBE is comparatively rare in Japanese individuals. RE and non-*H. pylori* infection were associated with a higher rate of progression to LSBE, considering the changes in exposures. *H. pylori* infection was associated with the prevention of the development of LSBE after the multivariable adjustment for potential confounders, including RE. In addition, the environment preventive of the development of LSBE persists for at least a few years after *H. pylori* eradication.

Time-dependent Cox models may have accurately estimated the relationship between time-varying RE and *H. pylori* status and the progression to LSBE. To date, a few studies have investigated the natural history of endoscopic or histological SSBE [9, 22, 29]. Among them, two studies reported the association between *H. pylori* infection/AG and SSBE elongation within 2- or 5.7-year periods [9, 22]. One study revealed that shortening of endoscopic SSBE tended to be associated with *H. pylori* and AG, although this was statistically nonsignificant [9]. Another study showed that the absence of AG was associated with the elongation of endoscopic SSBE [22]. These past studies were well designed; however, they did not take into account both the change in RE status and *H. pylori* eradication. These analyses may have underestimated the association between RE and *H. pylori* status and progression to LSBE. In our analysis, Cox models with baseline time-fixed covariates provided the attenuated HRs of RE and non-*H. pylori* infection for the progression to LSBE compared to time-dependent Cox models. In addition, the HR of smoking for the progression to LSBE was underestimated in Cox models by baseline time-fixed covariate. These results suggest that time-dependent Cox models were useful to accurately estimate the effects of time-varying exposures such as RE, *H. pylori* status, and smoking.

*H. pylori* infection was associated with the prevention of the development of LSBE in the time-dependent Cox models. This result is, at least in part, biologically plausible with respect to a decrease in gastric acid secretion. *H. pylori* infection causes gastric atrophy, decreasing gastric acid secretion [9]. The decrease in gastric acid secretion prevents RE, leading to the prevention of the development of LSBE. However, our study revealed that *H. pylori* infection was associated with the prevention of the development of LSBE after the multivariable adjustment for potential confounders, including RE. Indeed, the non-RE/*H. pylori*-positive patients were at lower risk of progression to LSBE than the non-RE/*H. pylori*-negative patients. Although the mechanism by which *H. pylori* infection suppressed the progression to LSBE independent of RE remains unclear, microbiota change in the esophagogastric junction and stomach may affect this suppression of the development of LSBE. Previous studies revealed that *H. pylori* infection reduces the diversity of bacterial microbiome in the stomach [30–32]. Furthermore, a prospective population-based study showed that *H. pylori* infection is involved in gastric microbial dysbiosis, which is associated with carcinogenesis [33]. Although the relationship between microbial dysbiosis and the development of LSBE has not been well studied, microbiota change in the esophagogastric junction and gastric mucosa caused by *H. pylori* infection may create an environment preventive of the development of LSBE.

The environment preventive of the development of LSBE persists for at least a few years after *H. pylori* eradication. Past studies have shown that *H. pylori* eradication increases gastric acid secretion, leading to an increased prevalence of RE [14, 34, 35]. This suggests that *H. pylori* eradication may also increase the rate of endoscopic SSBE elongation. Our study, however, failed to show such a tendency. Our results may indicate that *H. pylori* eradication may not increase the rate of progression to LSBE. However, the results should be interpreted with caution. First, it was uncertain whether the follow-up period was sufficient to assess the association between *H. pylori* eradication and progression to LSBE. Past studies have shown that the risk of developing RE increases as the period after *H. pylori* eradication is extended [35]. Because in many cases, SSBE extends to LSBE after developing RE, *H. pylori* eradication may promote the progression to LSBE with longer follow-up. Furthermore, the association between *H. pylori* eradication and progression to LSBE may differ depending on the degree of AG before eradication. Only among subjects with mild AG, *H. pylori* eradication may increase gastric acid secretion, leading to progression to LSBE. Long-term prospective cohort studies are expected that take into account the degree of AG at the time of *H. pylori* eradication.

The association between the other possible risk factors and progression to LSBE was almost consistent with the reported studies [9, 22, 29]. In our study, age, male sex, smoking, and HH were associated with the progression to LSBE. However, the relationship between the administration of PPI or H2RA and the progression to LSBE was different from that in the previous study [9]. In our study, the administration of PPI or H2RA was associated with the progression to LSBE. One possible explanation for this is that PPI/H2RA is confounded by severe RE or gastroesophageal reflux disease, which are risk factors of elongation of endoscopic SSBE. That is, subjects taking PPI or H2RA may have potentially severe RE or gastroesophageal reflux disease, resulting in progression to LSBE. However, such an association could not be demonstrated in our database. As our objective for the multivariable adjustment was not to assess the increase in risk with these medications, actively interpreting a coefficient of PPI/H2RA may be misleading [36].

Progression from endoscopic SSBE to LSBE is comparatively rare in Japanese individuals. A major reason for the low incidence of developing LSBE may be the different clinical criteria of SSBE. In the United States and Europe, intestinal metaplasia is needed for the diagnosis of SSBE; however, SSBE is diagnosed without histological confirmation of intestinal metaplasia in Japan and the United Kingdom [4, 37, 38]. Endoscopic SSBE in Japan may include more cases that are not diagnosed with BE according to the criteria of other countries. Indeed, there are far more subjects endoscopically diagnosed with SSBE in Japan than those histologically diagnosed with SSBE in other countries [39]. A substantial proportion of endoscopic SSBE cases in Japan may have lower malignant potential. In fact, in our study, only 34 subjects developed LSBE (1.0 per 1000 person-years). This result suggests that not all subjects with endoscopic SSBE should be followed-up closely. Accurate selection of patients with a high risk for developing LSBE is important, and follow-up of these high-risk patients should be prioritized. Absence of *H. pylori* infection may be a marker of microbiota, which may be associated with a relatively high risk of developing LSBE.

There were several limitations. First, the accurate endoscopic assessment of the length of the BE is difficult; therefore, its reproducibility is relatively low [40]. To minimize this problem, we evaluated only whether the length of circumferential Barrett epithelium was more than 3 cm or not (LSBE or SSBE) [29]. Additionally, all cases initially diagnosed with LSBE by endoscopy experts were reconfirmed by the most experienced endoscopy specialist. However, this endoscopic diagnosis of SSBE and LSBE may lead to bias. For example, our analysis may not detect cases with subtle elongation of BE and may underestimate the prevalence of the development of LSBE. Indeed, among the 7637 subjects with SSBE, only 34 developed LSBE (1.0 per 1000 person-years). Second, the criteria for BE in Japan differ from those in Western countries [37]. At present, the definition of BE has not been universally established. In the United States and most European countries, histological confirmation of intestinal metaplasia is necessary for the diagnosis of BE, unlike in the United Kingdom and Asian countries. In our study, LSBE and SSBE were endoscopically diagnosed without histological examinations using the Prague C & M criteria. A previous study suggested that endoscopic diagnosis of BE using the Prague C & M criteria is relatively reliable, especially in the case of LSBE [40], but differences in the definition of SSBE may affect the results. In fact, a large cross-sectional study in Japan showed that the clinical features of endoscopic SSBE differ from those of Western SSBE [8]. Therefore, the results of this study may not be applicable to patients with SSBE worldwide. Third, all subjects in our study voluntarily took these medical surveys. Most subjects were men with medium and high socioeconomic status. Additionally, subjects who did not undergo an additional health-check program after being diagnosed with SSBE were excluded. These may have led to a selection bias. Fourth, the frequency of medical surveys varied from subject to subject. Finally, the number of subjects who experienced the progression to LSBE was rather small. A large long-term study is expected in the future.

## Conclusions

We showed that progression to LSBE is comparatively rare in Japanese individuals. Non-RE and *H. pylori* infection was associated with a lower rate of progression to LSBE in a Japanese population, considering the changes in exposure. *H. pylori* infection was associated with the prevention of the development of LSBE irrespective of RE. In addition, the environment preventive of the development of LSBE persists for at least a few years after *H. pylori* eradication.

## Supplementary information

**Additional file 1 Supplemental Table 1**. Subject characteristics at baseline. **Supplemental Table 2**. Association between RE and *H. pylori* status and the progression to LSBE adjusted by baseline time-fixed covariate. **Supplemental Table 3**. Association between *H. pylori* status and the progression to LSBE adjusted by baseline time-fixed covariate.

## Data Availability

The datasets used and/or analyzed during the current study are available from the corresponding author on reasonable request.

## Abbreviations

BE: Barrett’s esophagus
EAC: Esophageal adenocarcinoma
SSBE: Short-segment Barrett’s esophagus
LSBE: Long-segment Barrett’s esophagus
RE: Reflux esophagitis
*H. pylori*: *Helicobacter pylori*
OR: Odds ratio
CI: Confidence interval
AG: Atrophic gastritis
PPI: Proton pump inhibitor
H2RA: Histamine H2-receptor antagonist
HRs: Hazard ratios
