# Supplementary material for: Relationship between time-varying status of reflux esophagitis and Helicobacter pylori and progression to long-segment Barrett’s esophagus: time-dependent Cox proportional-hazards analysis
Source: BMC Gastroenterol. 2020 Aug 15;20:270. doi: 10.1186/s12876-020-01418-5 (PMC7429870; doi:10.1186/s12876-020-01418-5)
Supplement: Supplementary file 1 — Additional file 1 Supplemental Table 1. Subject characteristics at baseline. Supplemental Table 2. Association between RE and H. pylori status and the progression to LSBE adjusted by baseline time-fixed covariate. Supplemental Table 3. Association between H. pylori status and the progression to LSBE adjusted by baseline time-fixed covariate. [file 12876_2020_1418_MOESM1_ESM.docx]

**Supplemental digital content**

**Supplemental Table 1**. Subject characteristics at baseline

|  |  | | Subjects  (n=7,637) |
| --- | --- | --- | --- |
| Age (years) (mean (SD)) | | | 50.6 (10.3) |
| Sex, men (%) | | | 6,272 (82.1) |
| Smoking (pack-years) (mean ± SD) | | | 13.0 (16.3) |
| Alcohol consumption | | |  |
|  | Nondrinker (<40 g/week) (%) | | 2,899 (38.0) |
|  | Light drinker (40-140 g/week) (%) | | 2,058 (27.0) |
|  | Moderate drinker (140-280 g/week) (%) | | 1,477 (19.3) |
|  | Heavy drinker (≥280 g/week) (%) | | 1,199 (15.7) |
| Hiatal hernia (% positive) | | | 2,147 (28.1) |
| Reflux esophagitis (% positive) | | | 1,426 (18.7) |
| Proton pump inhibitor or histamine H2-receptor antagonist; (% positive) | | | 177 (2.3) |
| *H. pylori* status | | |  |
|  | Absence of *H. pylori* (%) | | 4,067 (53.3) |
|  | *H. pylori* infection (%) | | 3,551 (46.5) |
|  |  | Current *H. pylori* infection (%) | 2,732 (35.8) |
|  |  | *H. pylori* eradication (%) | 819 (10.7) |
| Reflux esophagitis and *H. pylori* status pattern | | |  |
|  | Reflux esophagitis (-)/*H. pylori* (+) | | 3,176 (41.6) |
|  | Reflux esophagitis (-)/*H. pylori* (-) | | 3,020 (39.5) |
|  | Reflux esophagitis (+)/*H. pylori* (+) | | 375 (4.9) |
|  | Reflux esophagitis (+)/*H. pylori* (-) | | 1,047 (13.7) |

SD, standard deviation; *H. pylori*, *Helicobacter pylori*

**Supplemental Table 2**. Association between RE and *H. pylori* status and the progression to LSBE adjusted by baseline time-fixed covariate

|  | | HR* | 95% CI | | P-value |
| --- | --- | --- | --- | --- | --- |
| RE and *H. pylori* status pattern | | | |  |  |
|  | RE(-) /*H. pylori* (+) | 1.00 | Reference | | |
|  | RE(-) /*H. pylori* (-) | 2.29 | 0.83 | 6.30 | 0.11 |
|  | RE(+) /*H. pylori* (+) | 3.26 | 0.80 | 13.22 | 0.098 |
|  | RE(+) /*H. pylori* (-) | 5.59 | 2.04 | 15.36 | <0.001 |
| Male | | 1.17 | 0.39 | 3.55 | 0.78 |
| Age (year) | | 1.06 | 1.02 | 1.10 | 0.002 |
| Smoking (100 pack-year) | | 1.64 | 0.29 | 9.11 | 0.57 |
| Hiatal hernia | | 3.42 | 1.63 | 7.22 | 0.001 |
| PPI or H2RA | | 2.41 | 0.72 | 8.11 | 0.16 |

*Adjusted by baseline time-fixed covariate (RE and *H. pylori* status pattern, male, age, smoking,

hiatal hernia, and PPI or H2RA).

RE, reflux esophagitis; *H. pylori*, *Helicobacter pylori*; LSBE, long-segment Barrett’s esophagus;

HR, hazard ratio; CI, confidence interval; PPI, proton pump inhibitor; H2RA, histamine H2-receptor antagonist

**Supplemental Table 3**. Association between *H. pylori* status and the progression to LSBE adjusted by baseline time-fixed covariate

|  |  | Multivariable model 1 | | | |  | Multivariable model 2 | | | |
| --- | --- | --- | --- | --- | --- | --- | --- | --- | --- | --- |
|  | | HR* | 95% CI | | P-value |  | HR* | 95% CI | | P-value |
| *H. pylori* status | |  |  |  |  |  |  |  |  |  |
|  | Absence of *H. pylori* | 1.00 | Reference | | |  | 1.00 | Reference | | |
|  | *H. pylori* infection (Model 1) | 0.48 | 0.21 | 1.10 | 0.08 |  |  |  |  |  |
|  | Current *H. pylori* infection (Model 2) |  |  |  |  |  | 0.56 | 0.24 | 1.31 | 0.18 |
|  | *H. pylori* eradication (Model 2) |  |  |  |  |  | 0.23 | 0.03 | 1.75 | 0.16 |
| Male | | 1.18 | 0.39 | 3.56 | 0.78 |  | 1.17 | 0.39 | 3.55 | 0.78 |
| Age (year) | | 1.06 | 1.02 | 1.10 | 0.002 |  | 1.06 | 1.02 | 1.10 | 0.002 |
| Reflux esophagitis | | 2.61 | 1.29 | 5.26 | 0.007 |  | 2.63 | 1.30 | 5.31 | 0.007 |
| Smoking (100 pack-year) | | 1.66 | 0.30 | 9.25 | 0.56 |  | 1.65 | 0.29 | 9.20 | 0.57 |
| Hiatal hernia | | 3.45 | 1.63 | 7.26 | 0.001 |  | 3.44 | 1.63 | 7.24 | 0.001 |
| PPI or H2RA | | 2.40 | 0.71 | 8.08 | 0.16 |  | 2.42 | 0.72 | 8.16 | 0.15 |

* Adjusted by baseline time-fixed covariate (RE and *H. pylori* status pattern, male, age, smoking, hiatal hernia, and PPI or H2RA).

*H. pylori*, *Helicobacter pylori*; LSBE, long-segment Barrett’s esophagus; HR, hazard ratio; CI, confidence interval; PPI, proton pump inhibitor;

H2RA, histamine H2-receptor antagonist
